# Pituitary Hormones mRNA Abundance in the Mediterranean Sea Bass *Dicentrarchus labrax*: Seasonal Rhythms, Effects of Melatonin and Water Salinity

**DOI:** 10.3389/fphys.2021.774975

**Published:** 2021-12-15

**Authors:** Jack Falcón, Maria Jesus Herrero, Laura Gabriela Nisembaum, Esther Isorna, Elodie Peyric, Marilyn Beauchaud, Joël Attia, Denis Covès, Michael Fuentès, Maria Jesus Delgado, Laurence Besseau

**Affiliations:** ^1^Biologie des Organismes et Ecosystèmes Aquatiques (BOREA), MNHN, CNRS UMR 8067, SU, IRD 207, UCN, UA, Paris, France; ^2^Sorbonne Université, CNRS, Biologie Intégrative des Organismes Marins (BIOM), Banyuls-sur-Mer, France; ^3^Department of Genetics, Physiology and Microbiology, Complutense University of Madrid (UCM), Madrid, Spain; ^4^Equipe de Neuro-Ethologie Sensorielle, ENES/CRNL, CNRS UMR 5292, UMR-S 1028, Faculté des Sciences et Techniques, Université Jean-Monnet (UJM), Saint-Étienne, France; ^5^Station Ifremer de Palavas, Palavas-les-Flots, Nantes, France

**Keywords:** sea bass, pituitary, hormones, annual variations, melatonin, photoperiod, salinity

## Abstract

In fish, most hormonal productions of the pituitary gland display daily and/or seasonal rhythmic patterns under control by upstream regulators, including internal biological clocks. The pineal hormone melatonin, one main output of the clocks, acts at different levels of the neuroendocrine axis. Melatonin rhythmic production is synchronized mainly by photoperiod and temperature. Here we aimed at better understanding the role melatonin plays in regulating the pituitary hormonal productions in a species of scientific and economical interest, the euryhaline European sea bass *Dicentrarchus labrax*. We investigated the seasonal variations in mRNA abundance of pituitary hormones in two groups of fish raised one in sea water (*SW* fish), and one in brackish water (*BW* fish). The mRNA abundance of three melatonin receptors was also studied in the *SW* fish. Finally, we investigated the *in vitro* effects of melatonin or analogs on the mRNA abundance of pituitary hormones at two times of the year and after adaptation to different salinities. We found that (1) the reproductive hormones displayed similar mRNA seasonal profiles regardless of the fish origin, while (2) the other hormones exhibited different patterns in the *SW vs*. the *BW* fish. (3) The melatonin receptors mRNA abundance displayed seasonal variations in the *SW* fish. (4) Melatonin affected mRNA abundance of most of the pituitary hormones *in vitro*; (5) the responses to melatonin depended on its concentration, the month investigated and the salinity at which the fish were previously adapted. Our results suggest that the productions of the pituitary are a response to multiple factors from internal and external origin including melatonin. The variety of the responses described might reflect a high plasticity of the pituitary in a fish that faces multiple external conditions along its life characterized by marked daily and seasonal changes in photoperiod, temperature and salinity.

## Introduction

In vertebrates, the neuroendocrine regulations are controlled by the brain-pituitary (BP) axis. The hypothalamus receives information from external and internal origin, which is converted into messages that regulate the hormonal productions of the pituitary gland. The pituitary hormones then act on downstream peripheral targets, thus controlling functions as important as reproduction, growth, osmoregulation (aquatic vertebrates), feeding, lactation (mammals), pigmentation, immunity or stress. As for most functions of the organism, the neuroendocrine regulations display daily and/or annual rhythmic patterns (Ekström and Meissl, 1997; Cowan et al., 2017; Falcón and Zohar, 2018). The alternation of light (L) and darkness (D) over the 24 h cycle (photoperiod) plays a major role in synchronizing these rhythms, while other factors are also involved (*e.g*., temperature, water salinity, food availability, rainfall) (Falcón et al., 2010). Synchronization to these factors generally involves internal daily (circadian) and seasonal (circannual) clocks (Migaud et al., 2010).

In fish, virtually all cells possess the molecular machinery that makes a functional biological clock (Whitmore et al., 2000a,b), which constitute a network of more or less potent oscillators. In this network, the photosensitive pineal organ plays a pivotal role (Underwood, 1989; Falcón et al., 2007; Falcón and Zohar, 2018). It produces melatonin – a time-keeping molecule – in higher amounts at night than during day. The amplitude of the nocturnal increase depends on various inputs (*e.g.*, external temperature, internal neurotransmitters and neuromodulators). Previous studies indicated that melatonin may directly regulate the pituitary hormonal secretions: (1) Messenger RNA corresponding to melatonin receptors and/or melatonin binding sites have been identified in the pituitary of several fish species (Gaildrat and Falcón, 1999, 2000; Gaildrat et al., 2002; Park et al., 2006; Ikegami et al., 2009; Confente et al., 2010; Shin et al., 2011; Hong et al., 2014; Ciani et al., 2019; Sakai et al., 2019); (2) in isolated cultured pituitary glands melatonin modulates (i) the production of the 2nd messenger cyclic AMP (Gaildrat and Falcón, 1999, 2000; Falcón et al., 2003), (ii) the release of luteinizing hormone (LH) (Khan and Thomas, 1996), growth hormone (GH) and prolactin (PRL; Falcón et al., 2003), and (iii) the abundance of the clock genes mRNA *Cry1* and *Cry2* (Herrero and Lepesant, 2014). Melatonin acts also upstream the pituitary, as its receptors have been identified in the preoptic area (POA) and hypothalamus (Ekström and Vaněček, 1992; Davies et al., 1994; Oliveira et al., 2008; Ikegami et al., 2009; Herrera-Pérez et al., 2010), and modulates the secretion of gonadotropin releasing hormone (GnRH; Khan and Thomas, 1996; Sebert et al., 2008; Servili et al., 2013).

The role of melatonin as a neuroendocrine regulator in fish is no more questioned. However, a clear-cut picture is still missing perhaps because a sum of factors needs to be considered including the species investigated (and within the same species, the genetic diversity, sex, age, geographical location, previous history…), as well as the experimental conditions (*in vivo vs. in vitro*, photoperiod, temperature, salinity, time of day and time of year). Here we focused attention on the European sea bass – *Dicentrarchus labrax* – a euryhaline species of great economical interest in aquaculture. *D. labrax* is the matter of intensive molecular, physiological and behavioral investigations, which provide useful basic knowledge and molecular tools. Most of the studies on the sea bass have focussed on the BP-gonadal and BP-interrenal axis, including investigations on the daily and annual variations in melatonin and reproductive hormones plasma levels as well as in pituitary mRNA abundance of the latter (Rodriguez et al., 2001; Bayarri et al., 2004, 2009, Varsamos et al., 2006; Cowan et al., 2017; Sánchez-Vázquez et al., 2019). Here we investigated the annual variations in mRNA abundance corresponding to the three melatonin receptors cloned in the Mediterranean sea bass, and a range of pituitary hormones, including the reproductive hormones LH (or gonadotropin II, β subunit) and FSH (follicle-stimulating hormone or gonadotropin I, β subunit), as well as GH, PRL, POMC (proopiomelanocortin), SL (somatolactin) and TSH (thyroid stimulating hormone, β subunit). We also investigated *in vitro* the impact of melatonin and of melatonin analogs on the mRNA abundance of these pituitary hormones at two times of the year and in fish previously adapted to two different salinities.

## Animals, Materials and Methods

### Animals and Rearing Protocols

*Dicentrarchus labrax* used in this study originated from commercial aquaculture plants (Supplementary Figure 1A). Most of the experiments were performed with fish from “Méditerranée Pisciculture” [Salses-Le-Chateau, France; 42°50′ North (N), E2°55′ east (E)]. At the aquaculture plant the fish were reared outdoors, under natural conditions of photoperiod. The tanks were supplied with a mixture of seawater (from the Mediterranean Sea) and freshwater (from a local resurgence), so that the resulting salinity and temperature were maintained constant all year long (∼7‰ salinity and 16 ± 2°C, respectively). Random samplings indicated that most of the fish were males, in agreement with previous studies showing the sex ratio of farmed sea bass is highly biased, with a high proportion of males (ranging between 75 and 100%: Piferrer et al., 2005; Vandeputte et al., 2012, 2014, 2020), particularly at the water temperature at the “Méditerranée Pisciculture” facilities.

Upon arrival at the lab facilities of Banyuls-sur-Mer (France; 42°28′ N, 3°12′ E) the fish were placed for 3 weeks in 1 m^3^ indoors tanks (35 fish per tank) supplied with Mediterranean Sea water (∼37‰ salinity along the French Mediterranean coast) provided by an open circuit, at a flow rate of ∼400 l/h (Supplementary Figure 1B). In the experiments investigating the effects of salinity, the seawater was replaced by freshwater (4‰ salinity), all other conditions remaining similar [natural temperature and simulated natural photoperiod (light provided by fluorescent bulbs)] (Supplementary Figure 1B). To minimize stress induced by sampling, the fish were evenly distributed in different tanks, isolated one from each other by a double layer of thick black plastic curtains, so that the sampling was carried out in an alternate manner. Fish were fed *ad libitum*. Dark sampling was performed under dim red light. Mortality was close to 0 in the lab facilities. From now on these fish are named *BW* fish as they had been reared in brackish water before their arrival at the lab. As specified in Supplementary Figure 1, these fish were used for the *in vivo* (annual variations and acclimation to water salinities), and *in vitro* (melatonin challenges), experiments.

A second group of fish was made available to us, which originated from “Cannes aquaculture” (Cannes, France; 43°33′ N, 7°00′ E) and had been maintained for more than a year at the IFREMER station of Palavas-les-Flots (France; 43°31′ N, 3°55′ E). At the IFREMER station, they were placed in 10,000 l indoors round tanks, under simulated natural photoperiod (light provided by fluorescent bulbs) and natural temperature (see details below and Supplementary Figure 1A). Two major differences distinguished these fish from the *BW* fish described above: (1) they experienced seawater only (salinity ∼37‰) and (2) the same pool of fish was used from the beginning until the end of the experiment, so that they weighed 172 ± 37 g (mean ± S.E.) at the beginning, and 388 ± 77 g at the end, of the experiment (*i.e*., 12 months later). When brought to the lab the fish were kept in seawater, and were thus named *SW* fish (Supplementary Figure 1B); all other lab conditions were similar to those described above for the *BW* fish. These fish were used to investigate the annual variations in abundance of pituitary hormones and melatonin receptors mRNA (Supplementary Figure 1).

All experiments were performed according to the European Union regulations concerning the protection of experimental animals, and in agreement with the protocols approved by the Animal Care and Use Committee of the Observatoire Océanologique of Banyuls sur Mer (CNRS-UPMC; authorization # A-66-01-601).

### Daily and Annual Variations of Pituitary Hormones and Melatonin Receptors

The fish were sacrificed by decapitation every 4 h of a 24 h LD cycle (starting at 08:00) (Supplementary Figure 1C). The sampling was performed in April (13°C, water temperature), June (22°C), October (20°C), January (13°C) and March (13°C). After removal, the pituitary glands were dipped in RNAlater ^®^ (Applied Biosystems, Courtaboeuf, France). At each sampling time, 5 pools of 3 pituitary glands each were obtained for the *BW* fish. For technical reasons (size and number of fish available) only two 2 pools of 5 pituitary glands each were collected for the *SW* fish. The pools were frozen and kept at −80°C until mRNA extraction (as indicated below).

### Organ Culture and Pharmacological Treatments

After dissection, pituitary glands from *BW* fish (Supplementary Figure 1C) were cultured individually as detailed elsewhere (Falcón et al., 2003). Briefly, the pituitary glands were rinsed and cultured for 2 days in 750 μL of HEPES-buffered RPMI-1640 culture medium (one pituitary per well of a 24-well culture plate) at the temperature at which the fish had been acclimated. At noon of day 3, the pituitaries were challenged for 24 h with melatonin concentrations ranging from 10^–12^ to 10^–8^ M, which correspond to the plasma physiological concentrations measured during daytime and night-time in *D. labrax* (Garcia-Allegue et al., 2001; López-Olmeda et al., 2009). The effects of S20304 (10^–8^ M) and luzindole (10^–7^ M), respectively, a non-selective agonist and a non-selective antagonist at the mammalian melatonin receptors, were also investigated. At the end of the treatment, pools of 3 glands each were dipped in RNAlater ^®^ and stored at −80°C. The *in vitro* experiments investigating the effects of melatonin were carried out in February and August, *i.e*., during the reproductive and resting seasons, respectively. The experiments conducted in February were performed with pituitaries of fish adapted to salinities of 37 or 4‰ salinities, and those in August with pituitaries of the 37‰, salinity adapted fish. Data are expressed as the mean ± S.E.M. More details are provided in the figure legends and in Supplementary Figure 1.

### Total RNA Extraction, cDNA Synthesis, PCR and Real Time Quantitative PCR

Total RNA from the pituitary glands was extracted using the TRIZOL reagent (Life technologies SAS; Saint Aubin, France) according to the manufacturer’s instructions. The concentration and quality of the RNA was checked by spectrophotometry (Bio-photometer, Eppendorf, Le Pecq, France) and after migration in a MOPS gel. One μg of RNA was reversed transcribed using the Superscript Reverse transcriptase (Clontech, Saint-Germain-en-Laye, France). The cDNA obtained was stored at −20°C.

Real time quantitative PCR (qPCR) was performed using a Light Cycler 1.5 (Roche Diagnostics, Meylan, France) and the Light Cycler Fast Start DNA Master SYBR Green I kit (Roche Molecular Biochemicals, Meylan, France). The conditions of the reaction were 10 min at 95°C followed by 10 s at 95°C, 5 s at 60°C and 15 s at 72°C, for 30 cycles. The melting curves were analysed for each sample, to check that only a single sequence was amplified. They were obtained after increasing temperature progressively to 95°C, decreasing it to 65°C for 20 s, and finally increasing it to 95°C. Negative controls included cDNA replacement by water and the use of non-transcribed total RNA. In order to ensure the specificity of the amplification, some controls also included running the obtained PCR products in an agarose gel, followed by extraction, sub-cloning and sequencing (Sauzet et al., 2008). The 2^–ΔΔCt^ method was used to determine the relative mRNA abundance (Livak and Schmittgen, 2001), with the L17 ribosomal protein mRNA as the reference gene (Herrera-Pérez et al., 2011). The primers (Table 1) were designed using the Light Cycler Probe Design software (Roche, Meylan, France). Their efficiency was tested using serial dilutions of cDNA from pituitary extracts; the calibration curves exhibited slopes and efficiencies close to −3.32 and 100%, respectively.

**TABLE 1:** List of primers.

Hormone and accession number	Primers
Folliculo-Stimulating Hormone (FSH) AAN40506	F: CCACGAGGATCTGGTC R: TGTCTCCAGGAAAGCG
Luteinizing Hormone (LH) AAN40507	F: CTGGGAGCCACATCTTCC R: GACAGGGTCCTTAGTG
Thyroid Stimulating Hormone (TSH) KJ095101.1	F: TATACTCGGCCCACGC R: CACCTAGTTCCGTCCA
Prolactin (PRL) CAA55369	F: ACAAATAGCCAACAGAAGAG R: AGGATTACAAGGGGGTC
Growth Hormone (GH) AAD54100	F: ATCATCAGCCCCATCG R: TTGCCACCGTGAGGTA
Somatolactin (SL) AJ277390	F: CATCACCAAAGCCTTACCC R: GGCACATCATACTGGAATAGGC
Proopiomelanocortin (POMC) AY691808	F: GAGACACCGATCATCCC R: TGTACGTGCCATCCTT
MT1 melatonin receptor EU378918	F: GACAATCGGTCCCAAGC R: CCCTGCATCGTTAGAGC
MT2 melatonin receptor EU378919	F: TTGCAGTGGTTACCTACTGTTAT R: AGATGGCGAACAACACG
Mel1c melatonin receptor EU378920	F: GGCCTAAGCGTCATCG R: GTAGATGCGGGGATCG
L17 AF139590	F: CTGGCTTGCCTTTCTTGACT R: GAGGACGTGGTGGTTCATCT

### Statistical Analysis

Data were analysed using the one-way or two-way analysis of variance (ANOVA) followed by Holm-Sidak’s *post hoc* test for multiple comparisons. Individual means were compared using the Student’s *t* test (Two-tailed). Prism 6.01 (GraphPad™ software, La Jolla, CA, United States) was used to perform the statistics (level of significance set at *P* < 0.05) and plot the graphs.

### Chemicals

Unless otherwise specified, the products were from Sigma Aldrich (Saint Quentin Fallavier, France). S20304 was a kind gift of the ‘Servier Labs’ (Neuilly-sur-Seine, France).

## Results

### Annual Variations of Pituitary Hormones mRNA *in vivo*

Sampling started in April and ended in March of the following year. Relative abundance of mRNA from all the pituitary hormones investigated displayed significant annual variations.

**FIGURE 1:**
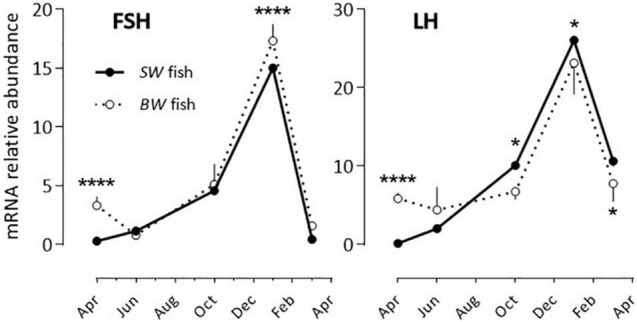
Relative annual variations in FSH and LH mRNA abundance in sea bass pituitaries *in vivo*. The pituitaries were collected from April until March of the following year. As detailed in the Materials and Methods section each value represents the mean ± S.E.M. (*n* = 6) of the values obtained from the 6 sampling hours (08:00, 12:00, 16:00, 20:00, 00:00, 04:00) of one light/dark cycle. At each sampling hour the sampling consisted of 5 pools of 3 pituitaries each (*BW* fish) or 2 pools of 5 pituitaries each (SW fish). All values are normalized to the value obtained from the *SW* fish in April (plotted as 1 in the Y axis). Two-way ANOVA indicated that the annual variations were statistically significant (Table 2) and the Holm-Sidak *post hoc* test was used for comparison of means between the *SW* and *BW* fish (Significant *: *P* < 0.05; ****: *P* < 0.0001).

FSH and LH: The profiles obtained were similar for both hormones and independent of the fish origin (*BW* or *SW* fish) (Figure 1 and Table 2). Levels were low from April to October, and then increased to reach a peak value in January and resume low levels in March.

**TABLE 2:** Two-way ANOVA data from Figure 1 (Annual variations in the abundance of mRNA of sea bass pituitary hormones).

mRNA	*F* value	*P* value <
**FSH**		
Interaction	9.67	0.0001
Month	865.20	0.0001
Salinity	45.83	0.0001
**LH**		
Interaction	14.24	0.0001
Month	267.70	0.0001
Salinity	0.18	ns
**GH**		
Interaction	6.22	0.0004
Month	22.36	0.0001
Salinity	361.6	0.0001
**POMC**		
Interaction	86.70	0.0001
Month	63.50	0.0001
Salinity	31.06	0.0001
**PRL**		
Interaction	170.3	0.0001
Month	17.70	0.0001
Salinity	67.95	0.0001
**SL**		
Interaction	23.75	0.0001
Month	246.50	0.0001
Salinity	7.02	0.0001
**TSH**		
Interaction	77.85	0.0001
Month	69.81	0.0001
Salinity	130.2	0.0001

**FIGURE 2:**
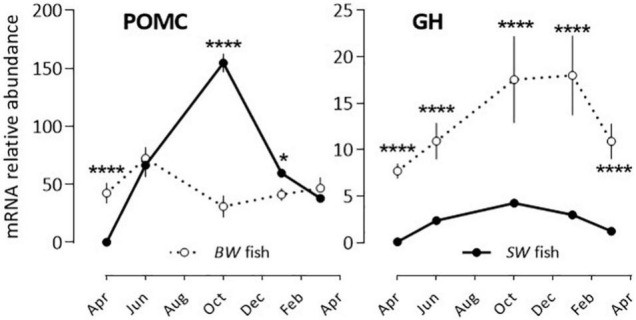
Relative annual variations in POMC and GH mRNA abundance in sea bass pituitaries *in vivo*. Same conditions as indicate in the legend of Figure 1. Mean ± S.E.M. (*n* = 6). Two-way ANOVA (Table 2) and Holm-Sidak’s *post hoc* test was used for comparison of means between the *SW* and *BW* fish (Significant *: *P* < 0.05; ****: *P* < 0.0001).

POMC and GH: POMC mRNA abundance displayed a bell-shape response (peak in October) in the *SW* fish, while no significant annual rhythm was seen in the *BW* fish (Figure 2 and Table 2). The opposite situation was observed with GH, which exhibited a U-shape profile in the *BW* fish (high values in October and January), and no significant variation in the *SW* fish (Figure 2 and Table 2).

**FIGURE 3:**
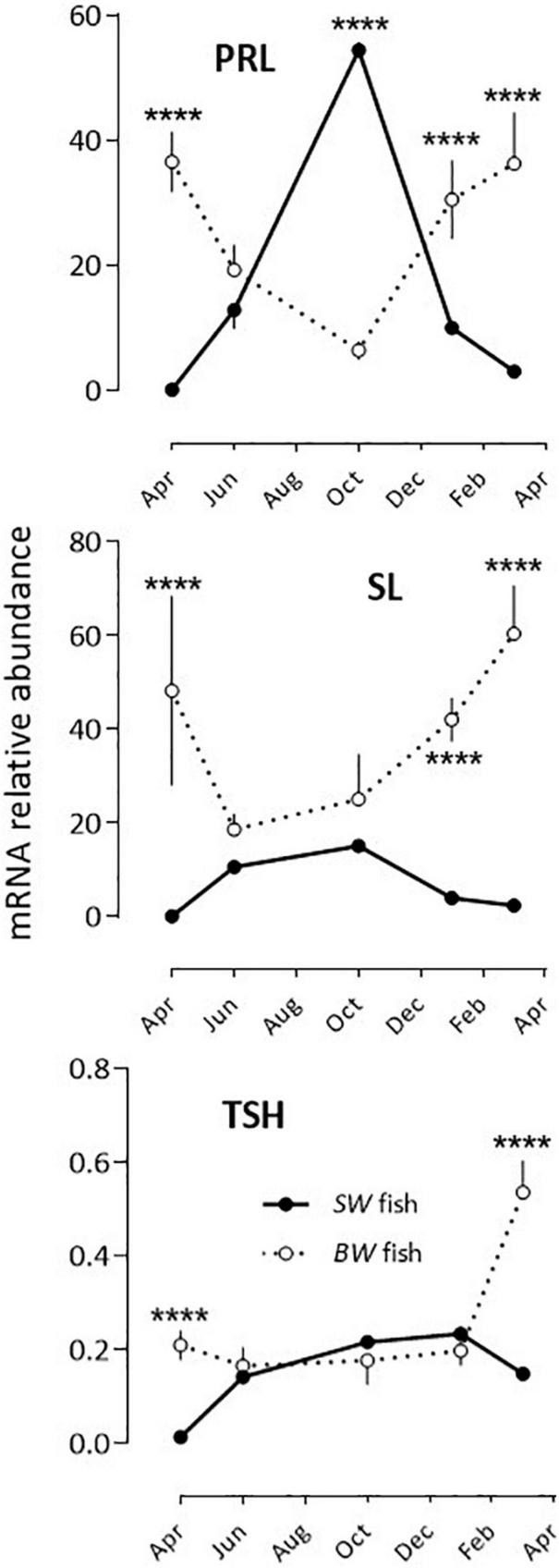
Relative annual variations in PRL, SL and TSH mRNA abundance in sea bass pituitaries *in vivo*. Same conditions as indicate in the legend of Figure 1. Mean ± S.E.M. (*n* = 6). Two-way ANOVA (Table 2) and Holm-Sidak’s *post hoc* test was used for comparison of means between the *SW* and *BW* fish (Significant *: *P* < 0.05; ****: *P* < 0.0001).

PRL, SL and TSH: Two situations were observed after comparing the *BW* to the *SW* fish as the annual variations displayed 180° out of phase profiles (Figure 3 and Table 2). In the *BW* fish, PRL mRNA relative abundance decreased significantly from April to October, and increased from October to March, while SL and TSH levels were high in March and low at the other months investigated.

### Daily and Annual Variations of Melatonin Receptors mRNA *in vivo*

**FIGURE 4:**
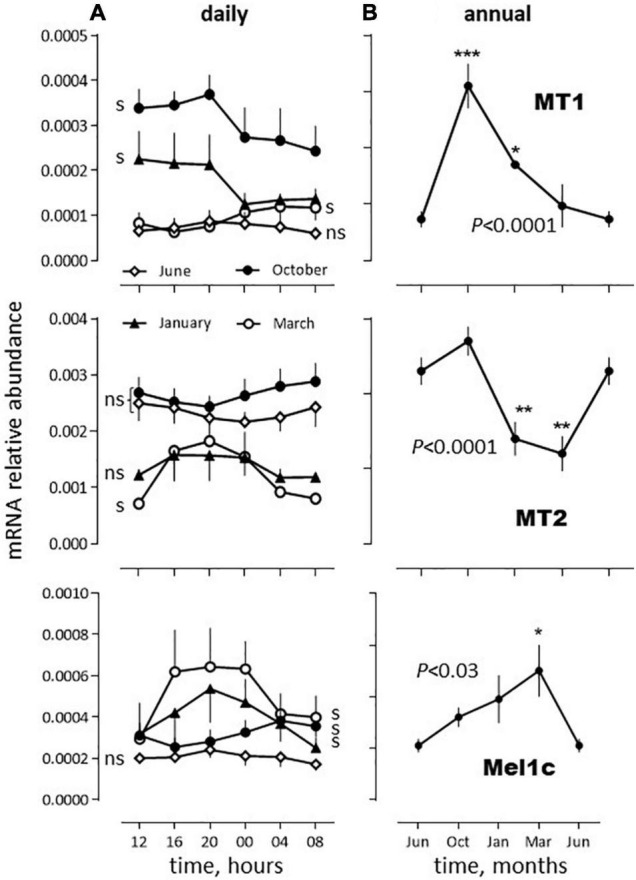
Relative daily **(A)** and annual **(B)** variations in mRNA abundance of sea bass melatonin receptors mRNA *in vivo*. The data were obtained from the same *SW* fish pituitaries extracts as used in Figures 1–3. (**A)** Daily variations: The moving average method was used because each sampling time was made of only 2 pools of 5 pituitaries each (as indicated in the Materials and Methods section). Thus, the plot at time *t*_*x*_ represents the mean of the values obtained at *t*_*x*–1_, *t*_*x*_ and *t*_*x+*1_. Mean ± S.E.M. (*n* = 6). Photoperiod was 14L_(07:00–21:00)_/10D (June), 12L_(08:00–20:00)_/12D, (October), 10L_(08:00–18:00)_/14D (January) and 12L_(08:00–20:00)_/12D (March). One-way ANOVA indicated *P* < 0.005 for MT1 in January, March and October, *P* < 0.0001 for MT2 in March and *P* < 0.01 for Mel1c in January, March and October; s: significant, ns: non-significant. **(B)** Annual variations: For each month the value obtained corresponds to the mean obtained at each sampling hour (in a) (mean ± S.E.M., *n* = 6). The June data are plotted twice. One way ANOVA level of significance is indicated in the graphs. Individual means are compared to the corresponding value in June using the Holm-Sidak’s *post hoc* test (Significant *P < 0.05; **P < 0.01; ***P < 0.005).

The three melatonin receptor subtypes cloned in the sea bass displayed daily variations in abundance in the *SW* fish, significant in March (MT1, MT2, Mel1c), January and October (MT1, Mel1c), but not in June (Figure 4A). They also displayed annual variations. The highest abundance was found in June and October for MT2, October for MT1 and March for Mel1c (Figure 4B).

### Impact of Salinity on Pituitary Hormones mRNA Abundance *in vivo*

**FIGURE 5:**
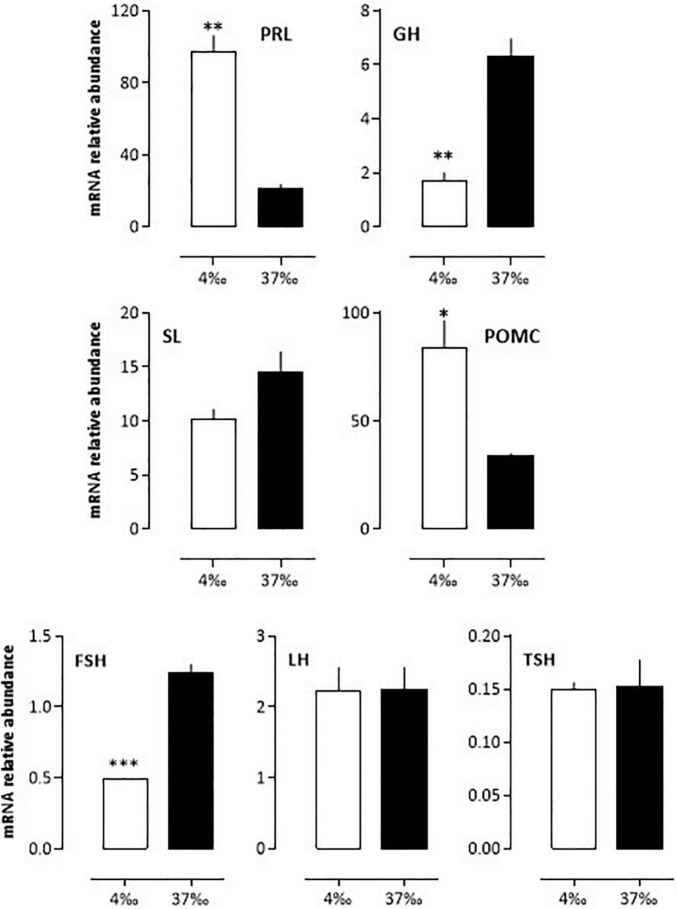
*In vivo* relative variations of mRNA abundance from bass pituitary hormones after a 3 weeks adaptation to 4 or 37‰ salinities. *BW* fish were maintained at the lab for 3 weeks in either sea- or freshwater as indicated in the Materials and Methods section. Mean ± S.E.M. (*n* = 5 samples, each sample containing a pool of 3 pituitary glands). Student’s *t*-test *P < 0.01, ^**^P < 0.001 and ^***^P < 0.0001.

After acclimation of the seabass to 4 or 37‰ salinities for 3 weeks in the lab, the mRNA abundance of pituitary hormones changed significantly for PRL (more than a 4-fold difference), GH, POMC and FSH (∼3-fold difference) (Figure 5). In the case of PRL and POMC, the levels were high under the 4‰ condition and low under the 37‰ condition, while the opposite held true for GH and FSH mRNA levels. SL levels were also 30% lower under the 4 *vs*. 37‰ condition, a difference not statistically significant. No difference was seen for LH and TSH (Figure 5).

### Effects of Melatonin on Pituitary Hormones mRNA Abundance *in vitro*

The effects of melatonin on the pituitary hormones mRNA levels were investigated in February and August, in *BW* fish acclimated for 3 weeks to a 37 or a 4‰ salinity (see experimental protocol design in Supplementary Figure 1).

**FIGURE 6:**
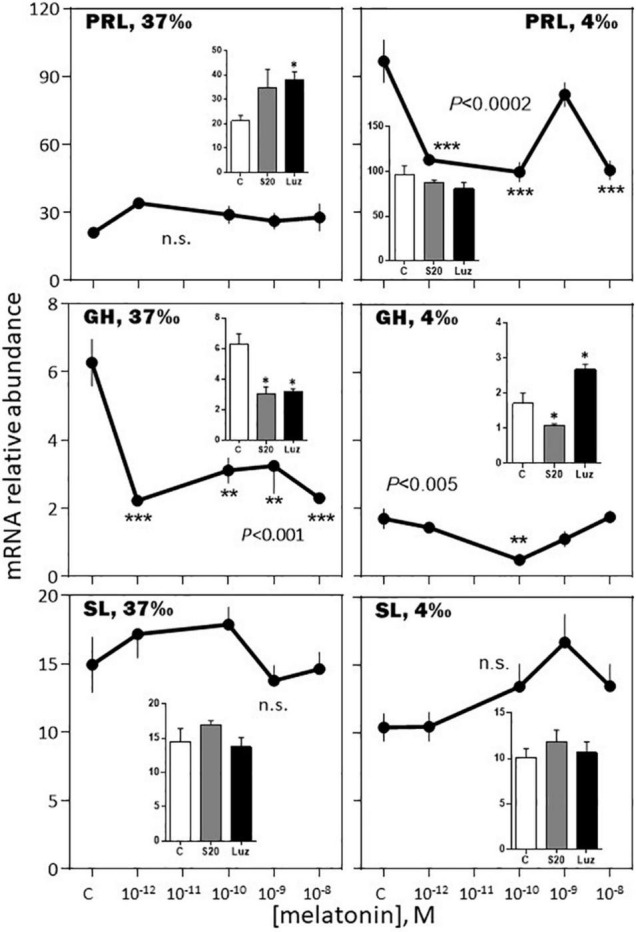
Effects of melatonin and melatonin analogs on PRL, GH and SL mRNA relative abundance *in vitro*. The experiment was performed in February. The *BW* fish were acclimated to either seawater (37‰; left column) or freshwater (4‰; right column) 3 weeks before sacrifice. The pituitaries were challenged for 12 h with the different concentrations of melatonin indicated in the abscissae (see Materials and Methods section for details). The controls (C in the abscissae) received an equivalent amount of solvent present at the highest melatonin concentration; it did not exceed 0.0001% in the final solution. Mean ± S.E.M. of *n* = 3 pools (containing 5 glands each). One-Way ANOVA (*P* gives the level of significance; n.s.: non-significant). Means were compared to the corresponding control using the Holm-Sidak’s *post hoc* test: *P < 0.05, **P < 0.005, ***P < 0.0005. The insets show the 12 h effects of two melatonin analogs S20304 (S20 at 10^– 8^ M) and luzindole (Luz at 10^– 7^ M). Mean ± S.E.M. of *n* = 3 pools (containing 5 glands each). Values are compared to the controls using the Student’s *t*-test at Significant *P < 0.01.

**FIGURE 7:**
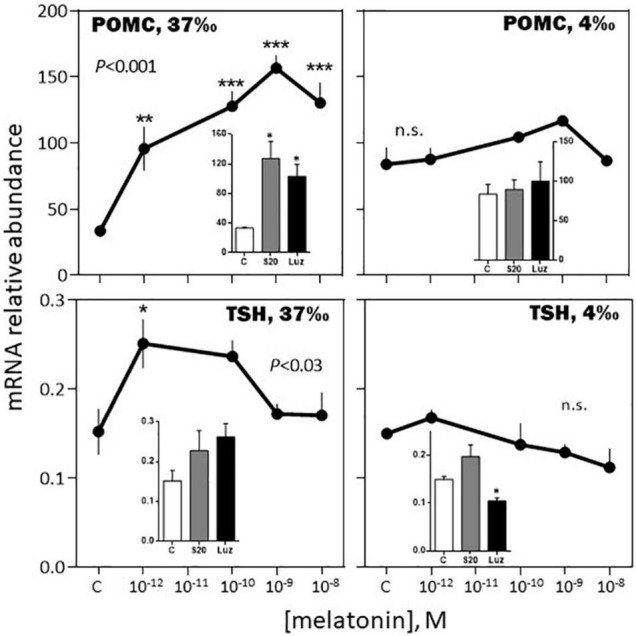
Effects of melatonin and melatonin analogs on POMC and TSH mRNA relative abundance *in vitro*. Mean ± S.E.M. of *n* = 3 pools (containing 5 glands each). One-Way ANOVA (*P* gives the level of significance; n.s.: non-significant). Means were compared to the corresponding control using the Holm-Sidak’s *post hoc* test: *P < 0.05, **P < 0.005, ***P < 0.0005. The insets show the 12 h effects of two melatonin analogs S20304 (S20 at 10^– 8^ M) and luzindole (Luz at 10^– 7^ M). Mean ± S.E.M. of *n* = 3 pools (containing 5 glands each). Values are compared to the controls using the Student’s *t*-test at *P* < 0.01. See Figure 6 for more details.

**FIGURE 8:**
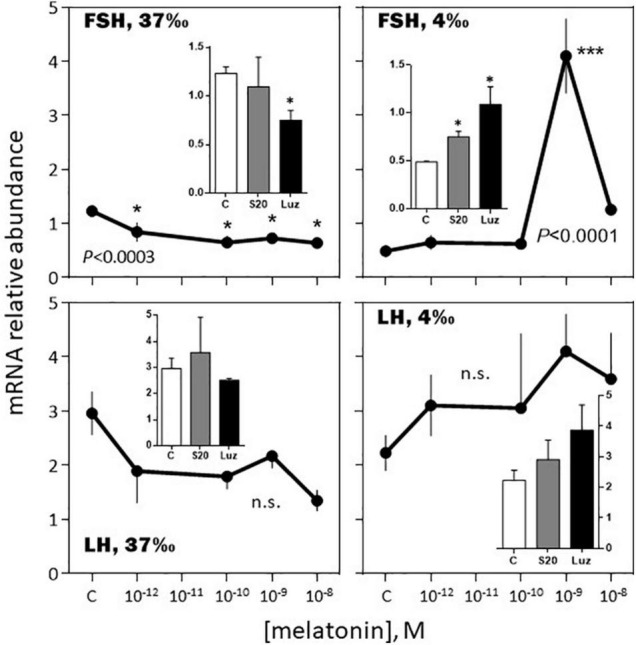
Effects of melatonin and melatonin analogs on FSH and LH mRNA relative abundance *in vitro*. Mean ± S.E.M. of *n* = 3 pools (containing 5 glands each). One-Way ANOVA (*P* gives the level of significance; n.s.: non-significant). Means were compared to the corresponding control using the Holm-Sidak’s *post hoc* test: *P < 0.05, ***P < 0.0005. The insets show the 12 h effects of two melatonin analogs S20304 (S20 at 10^– 8^ M) and luzindole (Luz at 10^– 7^ M). Mean ± S.E.M. of *n* = 3 pools (containing 5 glands each). Values are compared to the controls using the Student’s *t*-test at *P* < 0.01. See Figure 6 for more details.

*-February, 37‰ salinity*. Melatonin had no significant effect on PRL, SL and LH mRNA abundance (Figures 6–8). The hormone induced dramatic effects on GH and POMC, inhibitory for the former (3-fold inhibition at 10^–12^ M; Figure 6) and stimulatory for the latter (4-fold stimulation at 10^–9^ M; Figure 7). The FSH mRNA relative abundance was slightly inhibited by increasing concentrations of melatonin (Figure 8). The effect on TSH was complex as the stimulation observed at 10^–12^ and 10^–10^ M was reversed at 10^–9^ and 10^–8^ M (Figure 7). The effects were mimicked by the melatonin analogs S20304 and luzindole used at the concentration used (insets in Figures 6–8).

*-February, 4‰ salinity*. In the fish adapted to the 4‰ salinity, melatonin had no significant effects on SL, POMC, TSH and LH mRNA abundances (Figures 6–8). A v-shaped dose-response curve was obtained with GH (highest inhibition at 10^–10^ M of melatonin; Figure 6), while FSH mRNA levels were increased at the single concentration of 10^–9^ M (Figure 8). In a general manner, the effects were mimicked by the melatonin analogs S20304 and luzindole used at one single concentration (insets in Figures 6–8).

**FIGURE 9:**
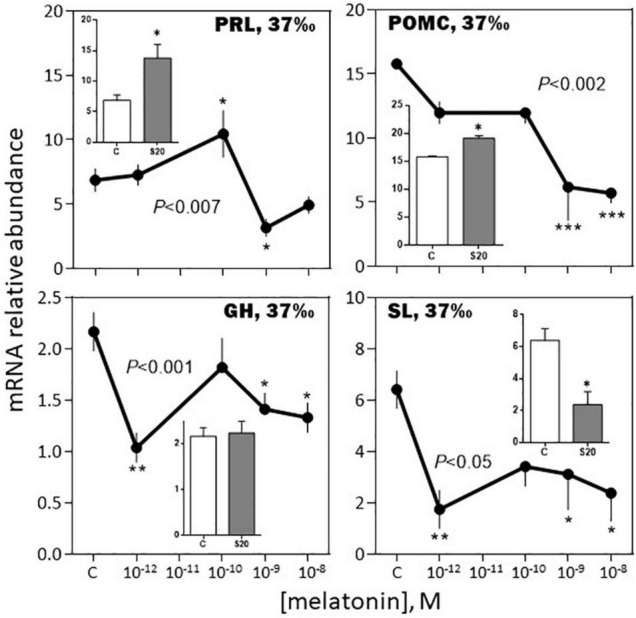
Effects of melatonin and melatonin analogs on PRL, POMC, GH and SL mRNA relative abundance *in vitro*. The experiment was performed in August using *BW* fish acclimated to a 37‰ salinity. Other conditions are as indicated in Figure 6 and in the Materials and Methods section. Mean ± S.E.M. of *n* = 5 pools (containing 3 glands each). One-Way ANOVA (*P* level of significance is indicated within the graph; n.s.: non-significant); values are compared to their respective control (C) using the Holm-Sidak’s *pos-hoc* test (*P < 0.05, **P < 0.005, ***P < 0.0005). The insets show the 12 h effects of the melatonin analogs S20304 (S20 at 10^– 8^ M). Mean ± S.E.M. of *n* = 5 pools (containing 3 glands each); values are compared to their respective control (C) using the Student’s *t*-test at *P < 0.01.

**FIGURE 10:**
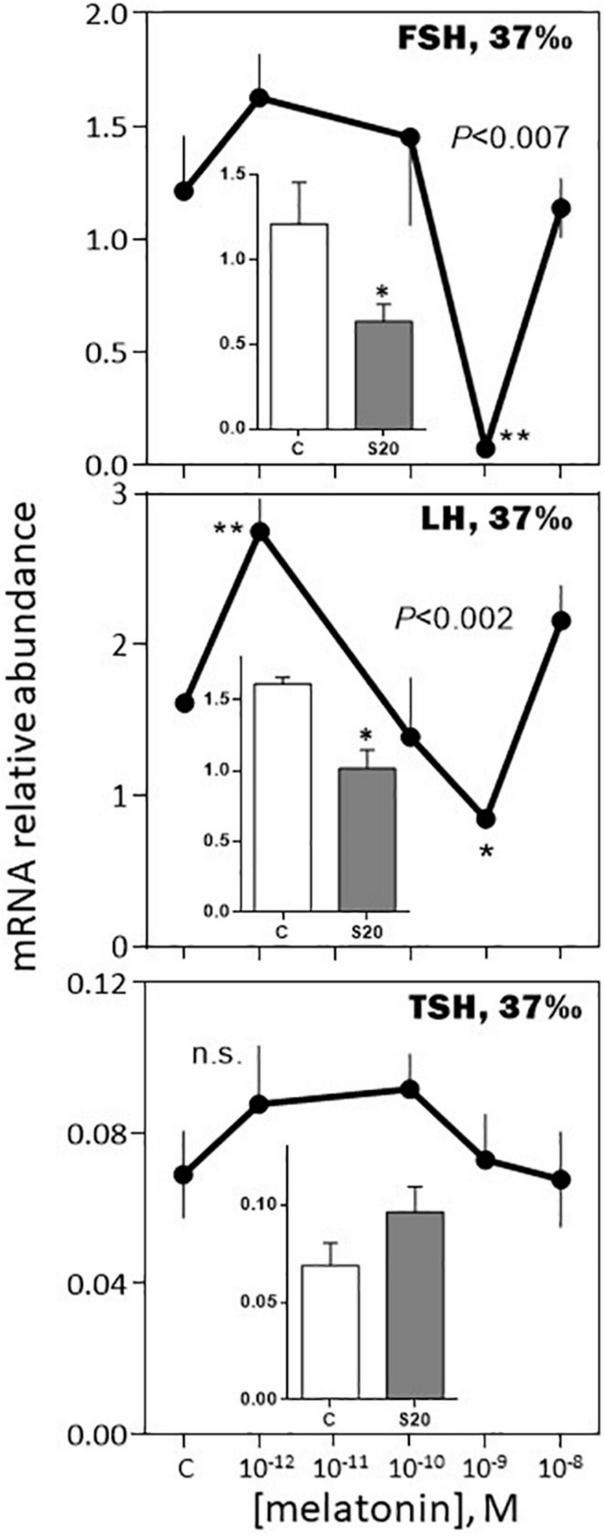
Effects of melatonin and melatonin analogs on FSH, LH and TSH mRNA relative abundance *in vitro*. Mean ± S.E.M. of *n* = 3 pools (containing 5 glands each). One-Way ANOVA (*P* gives the level of significance; n.s.: non-significant). Means were compared to the corresponding control using the Holm-Sidak’s *post hoc* test: *P < 0.05, **P < 0.005. The insets show the 12 h effects of two melatonin analogs S20304 (S20 at 10^– 8^ M) and luzindole (Luz at 10^– 7^ M). Mean ± S.E.M. of *n* = 3 pools (containing 5 glands each). Values are compared to the controls using the Student’s *t*-test at *P* < 0.01. See Figure 9 for more details.

*-August, 37‰ salinity*. While TSH mRNA abundance was not affected by melatonin, POMC, GH, SL mRNA relative levels were inhibited in a dose-dependent manner (Figures 9, 10). PRL and LH mRNA were also affected, however, the response was more complex, stimulatory at the low (10^–12^, 10^–10^ M), and inhibitory at the high (10^–9^ M), concentrations (Figures 9, 10). In a general manner, the effects of the melatonin agonist S20304 (10^–8^ M), were in agreement with those obtained with melatonin, except for POMC (inset Figure 9). For comparison the data obtained in February and August (at the salinity of 37‰) are plotted together in Supplementary Figures 2, 3.

## Discussion

### Annual Variations of Pituitary Hormones mRNA Abundance

Most of the studies reporting on the daily and annual variations of pituitary hormones focus on FSH and LH (Mateos et al., 2003; Cowan et al., 2017). Here we provide the first information on a panel of pituitary hormones in the European sea bass. Previous studies have shown a clear annual rhythm in the abundance of FSH and LH mRNA in the sea bass pituitary, with a peak occurrence in January/February, and lowest abundance from May to October (Mateos et al., 2003). This rhythm was paralleled by variations in the gonado-somatic index and plasma LH protein levels, in keeping with the annual rhythm of reproduction in this species (Kara, 1997; Alvarado et al., 2013). Our data agree fully with these previous findings. In addition, we found that similar profiles were obtained independent of the fish origin and previous rearing conditions, which brings strong support to the conclusion that the seasonal control of reproduction is a tightly locked photoperiod-dependent process in the European sea bass, as suggested earlier (Bayarri et al., 2004; Falcón et al., 2010).

A contrasting situation occurred with the other pituitary hormones, in which the annual rhythms of mRNA abundance differed in phase (TSH, SL, PRL) or amplitude (GH, POMC), in the *SW vs*. *BW* fish. The reason for these differences could lie in the fact that the fish originated from two different locations and had different historical backgrounds. The *BW* fish originated from distinct fertilization processes, and had all similar standard commercial sale size at each sampling month. In contrast, the *SW* fish were all from a single pool of fish that were followed during whole duration of the experiment. In addition, the *SW* fish were raised in a 37‰ salinity all life on, while the *BW* fish experienced a transition from a ∼7‰ to a ∼37‰ salinity 3 weeks before sacrifice. The differences may have impacted differently PRL, SL, GH, TSH, and POMC. These hormones have pleiotropic effects [control of skin pigmentation (POMC, SL), immunity and stress (POMC, GH, PRL), food intake (POMC, GH), growth (GH, TSH), osmotic regulation (GH, PRL), sexual maturation (GH, PRL, TSH)], and this involves a complex network of interactions within the pituitary, as well as the whole neuroendocrine loops (Agustsson and Bjornsson, 2000; Harris and Bird, 2000; Boeuf and Falcón, 2001; Bhandari et al., 2003; Deane and Woo, 2009; Laiz-Carrión et al., 2009; Blanco, 2020); in this regard, their regulation implies more flexibility than is the case for the reproductive hormones.

Part of the annual life cycle of the European sea bass involves salinity changes, which needs rapid adaptive neuroendocrine responses. We indeed observed that a 3 weeks adaptation to either low (4‰) or high (37‰) salinity impacted mRNA levels of GH, FSH, PRL, and POMC, while those of LH, SL, and TSH remained unaffected. In the case of PRL, POMC, SL, and GH, our data agree with those obtained in this (Varsamos et al., 2006) and other (Laiz-Carrión et al., 2009; Lu et al., 2016; Yamaguchi et al., 2016; Yuan et al., 2017; Liu et al., 2020; Vargas-Chacoff et al., 2021) fish species maintained under low or high salinities. They are also in line with the known roles of these hormones, essential in the processes of acclimation to different salinities (PRL, SL, GH, or rather the GH/IGF-I axis); both, PRL and GH/IGF-I interact with cortisol to control ionic balance (Takei and McCormick, 2013). The production of cortisol by the interrenal gland is controlled by ACTH (adrenocorticotropic hormone), a cleavage product of POMC. Here we found that POMC mRNA levels were higher under the freshwater than under the seawater condition, as observed in the ayu, an anadromous species (Lu et al., 2016), but in contrast to the situation in the tilapia, mainly a fresh water fish (Aruna et al., 2015). The case of SL is interesting as we found a slight non-significant increase in mRNA relative abundance, as is the case in the gilthead seabream (Laiz-Carrión et al., 2009); however, in the latter intermediate salinities (12‰) induced a dramatic decrease in SL mRNA compared to the low and high salinities (6 and 38‰, respectively), which highlights the complexity of the mechanisms studied.

### Daily and Annual Variations of Melatonin Receptors mRNA Abundance in the Pituitary

Melatonin receptors have been identified in the pituitary of several fish species (Gaildrat and Falcón, 1999, 2000; Gaildrat et al., 2002; Sauzet et al., 2008; Ikegami et al., 2009; Confente et al., 2010; Hong et al., 2014; Ciani et al., 2019; Sakai et al., 2019). Three subtypes have been identified in the sea bass (MT1, MT2, Mel1c), MT2 displaying the higher relative abundance in the pituitary (Sauzet et al., 2008; Herrera-Pérez et al., 2010; and this study). They also exhibited daily and seasonal variations as reported earlier for both, mRNA relative abundance and melatonin binding sites, in the pituitary and brain of other fish species (Falcón et al., 1996; Gaildrat et al., 1998; Amano et al., 2003; Park et al., 2006; Bayarri et al., 2010; Confente et al., 2010; Ciani et al., 2019). These variations are controlled by circadian clocks in the pike (Gaildrat et al., 1998) and circannual clocks in the European sea bass (Bayarri et al., 2010) as they were observed under constant conditions. Thus, a good understanding of the time-dependent effects of melatonin (as discussed below) requires that not only its cyclic production, but also the rhythmic availability and affinity of its receptors to be considered.

### Melatonin Effects on Pituitary Hormones

The current study supports previous investigations indicating melatonin treatment impacts LH, GH, and PRL productions in fish (Khan and Thomas, 1996; Falcón et al., 2003), and extends to POMC, SL, FSH, and TSH the range of hormones affected. In most cases, the conclusions were supported by the observed effects of the melatonin receptor agonist S20304 and antagonist luzindole. As characterized in mammals, luzindole is supposed to be an antagonist. However, paradoxical effects as the ones described in this study have been reported. In rats, luzindole may also act as a partial agonist at the MT2 receptor subtype (Legros et al., 2020) or exert direct proper effects (*i.e*., not mediated through melatonin receptors) on plasma membrane K^+^ currents (Zhou et al., 2003) or [Ca^2+^]_*i*_ stores (Estaras et al., 2020). In the medaka luzindole acted as a partial antagonist or a full agonist depending on the receptor subtype (Sakai et al., 2019). It has been emphasized that “*ligands that target melatonin receptor subtypes in one species does not have the same affinity and specificity in another species*” (ViviD and Bentley, 2018), which might be particularly true in fish, in which the receptors display proper structural and functional characteristics different from those of mammals.

In brief, the responses to the melatonin challenges appeared complex, depending for each hormone on several factors, including dose, time of year and previous adaptation to salinity.

#### The *in vitro* Response to Melatonin Depended on the Month of the Year

Melatonin had a moderate inhibitory effect on FSH and no effect on LH mRNA abundance in February. In August, the effect on FSH was amplified at the nanomolar concentration of melatonin, while LH mRNA displayed a biphasic response, stimulatory at the picomolar, and inhibitory at the nanomolar, concentrations of melatonin. Considering the sea bass annual reproductive cycle (Kara, 1997; Alvarado et al., 2013), these data are consistent with the idea that melatonin contributes to exert an inhibitory role on reproduction during the resting phase.

A novel finding of this study is the reported seasonal dependent impact of melatonin on the other hormones investigated. Strong in February (up to a 5-fold increase at 10^–9^ M melatonin), the increase in POMC mRNA relative abundance was no more observed in August. Similarly, the bell-shaped biphasic response of TSH was reduced in amplitude in August, compared to February. A reverse situation was observed for PRL and SL mRNA levels, no effect being seen in February, compared to an inhibitory response in August. Finally, the melatonin induced reduction of GH mRNA levels was less pronounced in August compared to February. We conclude that in the sea bass melatonin impacts all the pituitary hormones and the effects depend on the dose and month of the year. More studies at other months of the year are needed to get a more complete picture.

#### The *in vitro* Responses to Melatonin Depended on the Previous Fish Adaptation to Salinity

Previous investigations indicated that salinity changes affect both, melatonin production and melatonin binding sites in fish (Gern et al., 1984; Kleszczynska et al., 2006; López-Patiño et al., 2011), including in the Mediterranean sea bass, in which plasma melatonin levels, retina and brain maximal 2-[^125^I]-iodomelatonin binding (Bmax) were higher under low *vs*. high salinity adaptation. We have indication that the effects depend on the previous duration of adaption of the fish to a given salinity, and that this also affects the affinity (k_*D*_) of the binding sites for melatonin (authors’ unpublished data). These observations together with those discussed above concerning the modulation by salinity of mRNA levels of PRL, GH, TSH or POMC before the melatonin challenge, might in part explain that melatonin treatment resulted in different effects in the 4 *vs*. 37‰ acclimated fish. Compared to the 37‰ acclimated fish, in those acclimated to the 4‰ salinity the amplitudes of the dose-dependent responses to melatonin were dramatically reduced (POMC and GH), no more observed (TSH), or reversed (FSH). Whatever the situation, we conclude that melatonin effects on the pituitary depend highly on the salinity at which the fish had previously been adapted.

## Conclusion

This study on the European sea bass brings important new information on the control of neuroendocrine regulations in fish: (**1**) all the pituitary hormones investigated displayed annual variations, bringing together and in a single species, previous fragmentary information. (**2**) The pattern of the FSH and LH annual variations is robust, independent of the year investigated, fish origin and historical background, including previous salinity conditions. This is a strong support to the idea that the control of the annual cycle of reproduction is a robust and tightly regulated process where photoperiod is likely to play a major role; whether this involves a circannual clock remains an open question. **(3**) The other pituitary hormones (POMC, GH, PRL, SL, and TSH) deviate from this strictly controlled pattern as they displayed more flexible profiles depending on whether the fish had been reared under brackish or sea water. (**4**) Melatonin has pleiotropic effects in the pituitary of the European sea bass: we confirm, at the mRNA level, previously identified effects of melatonin on LH, GH and PRL proteins, and extend to FSH, POMC, SL, and TSH the range of hormones modulated by melatonin. At this stage it is not yet possible to determine whether the effects are direct and/or indirect. The cellular localization of the different melatonin receptor subtypes will help identifying the pituitary cellular targets. Also, more investigations are needed in order to determine whether the effects on mRNA are followed or not by effects at the level of the corresponding proteins for each of the hormones investigated. (**5**) The *in vitro* effects of melatonin depended on the month investigated as described before in trout (Falcón et al., 2003); this is in line with previous *in vivo* studies investigating the effects of pinealectomy on fish reproduction (Falcón et al., 2010; ViviD and Bentley, 2018). This highlights the necessity to run seasonal investigations when studying the effects of melatonin on neuroendocrine regulations, whether *in vivo* or *in vitro*. The effects depend on the daily and annual patterns of both, the circulating levels of the hormone as well as the abundance and affinity of its receptor subtypes. (**6**) Salinity appears as an important factor to consider when studying neuroendocrine responses in euryhaline species. Salinity modifies basal pituitary productions and melatonin plasma levels, as well as abundance of melatonin receptors. We believe the European sea bass pituitary exemplifies the statement that “*countless endogenous and plastic physiological factors, as well as the perception and transduction of predictable and unpredictable environmental cues, coordinate*… *to adapt to ever-changing environments”* (ViviD and Bentley, 2018). This is of crucial importance; it probably explains the observed versatility concerning the responses to melatonin from a species to another or even within the same species as evidenced here, making generalizations extremely difficult.

## Data Availability Statement

The original contributions presented in the study are included in the article/Supplementary Material, further inquiries can be directed to the corresponding author.

## Ethics Statement

The animal study was reviewed and approved by CNRS-UPMC; authorization #A-66-01-601.

## Author Contributions

JF: project coordinator, search for funds, experimental design, experiments, data analysis, writing, training students, and submission. MH: experiments, data analysis, and reading proofs. LN: experiments, data analysis, and reading proofs. EI: experiments and data analysis. EP: experiments. MB: experimental design, experiments, and reading proofs. JA: statistical analysis. DC: fish maintenance. MF: fish maintenance and samplings. MD: search for funds, students training, and reading proofs. LB: students training and reading proofs. All authors contributed to the article and approved the submitted version.

## Conflict of Interest

The authors declare that the research was conducted in the absence of any commercial or financial relationships that could be construed as a potential conflict of interest.

## Publisher’s Note

All claims expressed in this article are solely those of the authors and do not necessarily represent those of their affiliated organizations, or those of the publisher, the editors and the reviewers. Any product that may be evaluated in this article, or claim that may be made by its manufacturer, is not guaranteed or endorsed by the publisher.

## Abbreviations

BW: brackish water
D: dark
FW: freshwater
FSH: follicle-stimulating hormone or gonadotropin I- β
GH: growth hormone
L: light
LH: luteinizing hormone or gonadotropin II- β
PRL: prolactin
POMC: proopiomelanocortin
qPCR: real time quantitative PCR (qPCR)
SW: sea water
SL: somatolactin
TSH: thyroid stimulating hormone or thyrotropin β.
