# Supplementary figures and images for: Pituitary Hormones mRNA Abundance in the Mediterranean Sea Bass Dicentrarchus labrax: Seasonal Rhythms, Effects of Melatonin and Water Salinity
Source: Front Physiol. 2021 Dec 15;12:774975. doi: 10.3389/fphys.2021.774975 (PMC8715012; doi:10.3389/fphys.2021.774975)

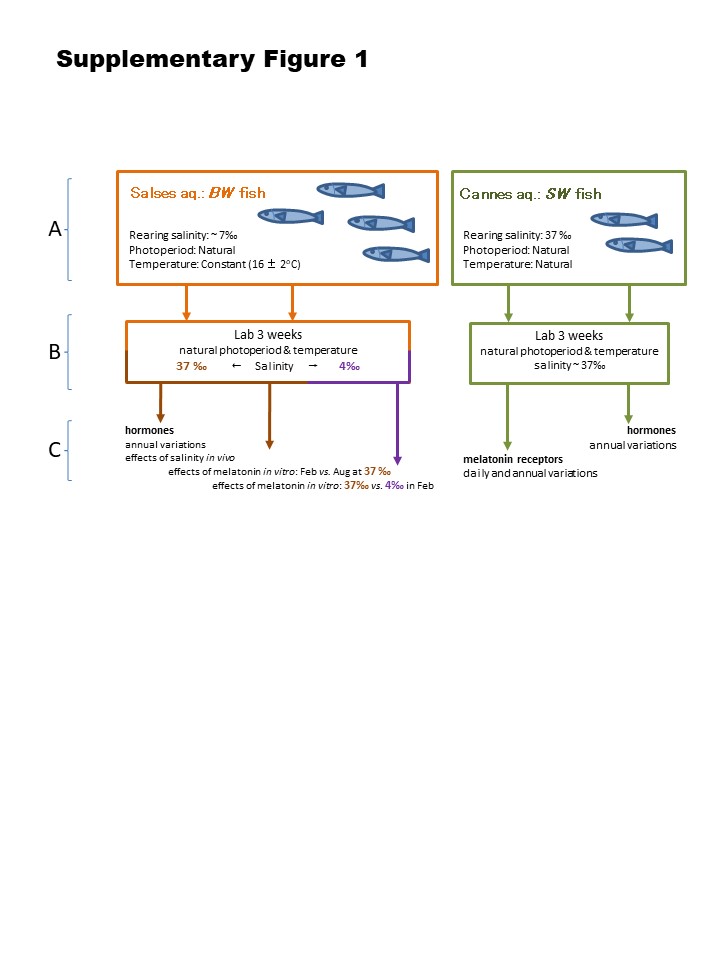

Supplement: Supplementary file 1 [file Image_1.JPEG]

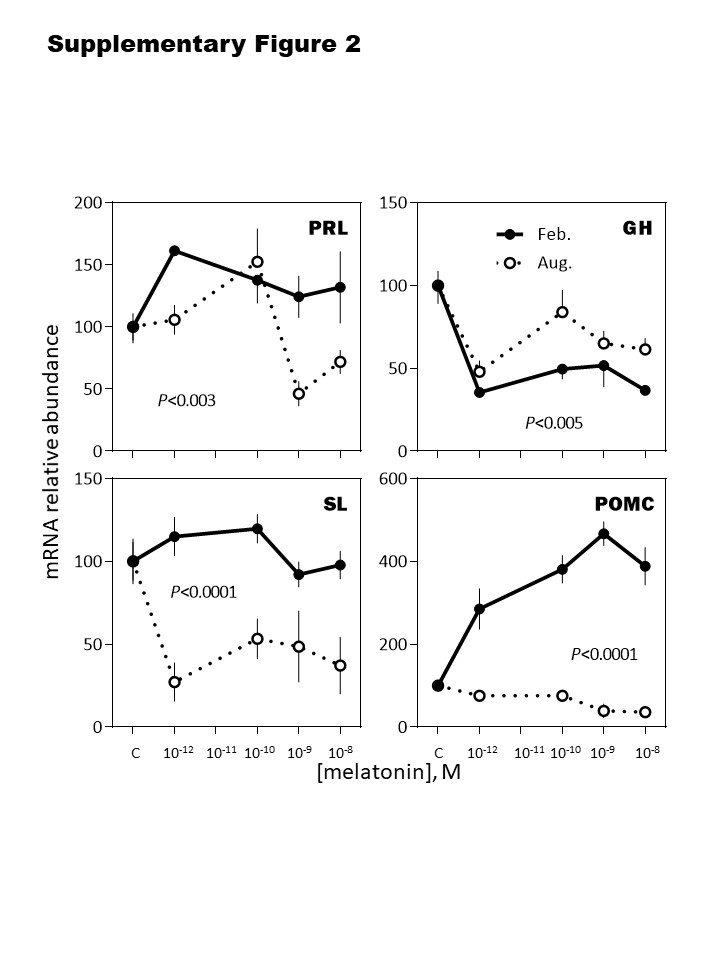

Supplement: Supplementary file 2 [file Image_2.JPEG]

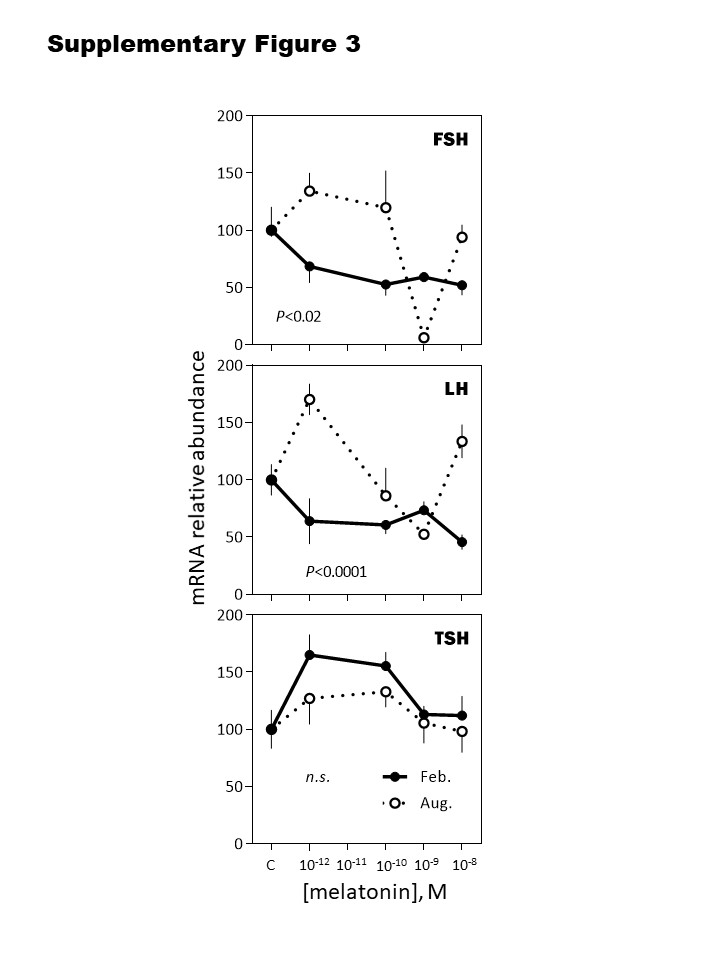

Supplement: Supplementary file 3 [file Image_3.JPEG]
