# Supplementary material for: Pituitary Hormones mRNA Abundance in the Mediterranean Sea Bass Dicentrarchus labrax: Seasonal Rhythms, Effects of Melatonin and Water Salinity
Source: Front Physiol. 2021 Dec 15;12:774975. doi: 10.3389/fphys.2021.774975 (PMC8715012; doi:10.3389/fphys.2021.774975)
Supplement: Supplementary file 4 [file Table_1.docx]

**SUPPLEMENTARY SECTION**

**Supplementary figures captions**

**Supplementary figure 1**. Schematic presentation of the experimental protocol using the fish reared in brackish (*BW*) sea (*SW*) water. Step **A**. Fish origin and rearing conditions. The *BW* fish were obtained directly from the Salses aquaculture (Aq.) plant each time they were needed for the experiment. The *SW* fish from Cannes Aq. had first been transferred at the IFREMER station of Palavas-Les-Flots, where they have been maintained for a yearlong under the conditions indicated. From there, both the *BW* and *SW* fish followed step B. Step **B**. Acclimation in the lab facilities. Step **C**. Experimental protocols.

**Supplementary** **figures 2 and 3. Effects of melatonin on mRNA relative abundance of pituitary hormones *in vitro***. The experiments were performed at two times of the year, February and August, using *BW* fish adapted to seawater (37‰) salinity for 3 weeks before sacrifice. The data are replotted from those presented in figures 6-10. See the corresponding legends for more details. For each curve the values are normalized to their respective control (100 % value). Mean ± S.E.M., n = 3 samples (each containing a pool of 5 pituitary glands). The results of the two-way ANOVA analyses are provided in Supplementary Table 1.

**Supplementary table 1. Two way ANOVA analysis of the data presented in supplementary figures 2 and 3.**

| **mRNA** | **F value** | ***P* value <** |
| --- | --- | --- |
| **FSH** | | |
| **Interaction** | 5.89 | 0.003 |
| **Melatonin** | 7.20 | 0.001 |
| **Month** | 6.69 | 0.02 |
| **LH** | | |
| **Interaction** | 8.88 | 0.0003 |
| **Melatonin** | 5.31 | 0.005 |
| **Month** | 22.87 | 0.0001 |
| **GH** | | |
| **Interaction** | 1.19 | n.s. |
| **Melatonin** | 14.27 | 0.0001 |
| **Month** | 10.2 | 0.004 |
| **POMC** | | |
| **Interaction** | 20.08 | 0.0001 |
| **Melatonin** | 10.34 | 0.0001 |
| **Month** | 247.4 | 0.0001 |
| **PRL** | | |
| **Interaction** | 2.99 | 0.004 |
| **Melatonin** | 4.54 | 0.009 |
| **Month** | 11.57 | 0.003 |
| **SL** | | |
| **Interaction** | 3.23 | 0.04 |
| **Melatonin** | 2.28 | n.s. |
| **Month** | 39.73 | 0.0001 |
| **TSH** | | |
| **Interaction** | 0.39 | n.s. |
| **Melatonin** | 3.72 | 0.02 |
| **Month** | 2.48 | n.s. |
